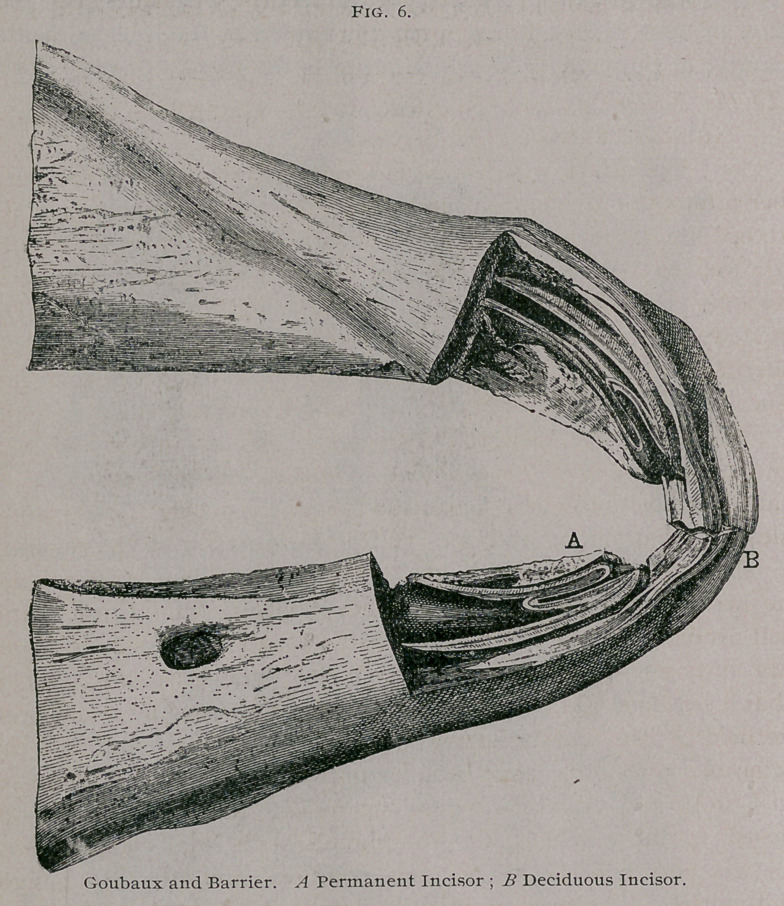# Age of the Horse, Ox, Dog, and Other Domestic Animals

**Published:** 1890-01

**Authors:** R. S. Huidekoper

**Affiliations:** Veterinarian


### AGE OF THE HORSE, OX, DOG, AND OTHER DOMESTICATED ANIMALS.

BY R. S. HUIDEKOPER, M.D., VETERINARIAN.[1]

### PREFACE.

AGE is defined by Webster as "the whole duration of a being," or "that part of the duration of a being which is between its beginning and any given time." The age of the domesticated animals is a matter of great importance in agricultural commerce, as in the limited period during which each of them is individually

[1] Due credit will be given in this article to all authors from whom quotations are made. A large portion of the facts and numerous plates, especially in regard to the dentition of the horse are taken directly from *L'Exterieur du Cheval*, by *MM. Goubaux* and *Barrier*, whose work was so well done that the author has found place for little revision.

useful, a comparatively short time diminishes greatly the extent of usefulness to which each can be put, and consequently lowers its value as an investment. The means which enable us to judge the age of an animal, are based upon certain anatomical and physiological changes which occur in the course of the development ot the new born to the adult animal, and in the deterioration of it from its period of perfection to the decrepitude of its last years. Age can be divided into three periods : First, *juvenile age or the period of growth*, which extends from the birth of the animal to its full development, during which it is gaining in size, in strength, in intelligence, and is constantly increasing in its qualities and value. Second, *adult age, or the stationary period ;* in this the animal is at its best ; it has every organ complete in size and function, and working in perfect harmony with the rest of the body. Its intellect has the most complete control over its perfectly organized muscles, digestive tract and other apparatuses, and it is capable of its best and greatest work. Third, *senility, or old age*, the period of deterioration. During this period the animal may have the strength of previous years, it may be capable of as great efforts, and from experience may even display greater intelligence, for specific purposes the animal may at this time be capable of rendering even greater service than it could have given when younger, but its efforts now demand a waste of tissues no longer capable of rapid repair, and continual drains on its organs diminishes them in size and function, and the whole animal economy undergoes a steady deterioration in usefulness and value.

The veterinarian is the expert referee in all questions of age, and any addition to our knowledge in regard to it, needs no apology.

If animals lived a perfectly equable life, the changes which take place in their form and character would be regular and easy to appreciate, but, from over work, from the alterations produced by excessive food, want of food, or irritant substances, from the effects of variable surroundings, at times trying on the constitution, at other times rendering the external tissues delicate and sensitive to exposure, the wear and tear of the tissues is irregular. Excessive work in one animal, or want of food in another, open easy avenues for the ravages of time which would not show on one which was better nourished and better cared for. The well bred colt training ver fast miles from a yearling, or the great draught horse hauling a load of several tons in our large cities, demand excess of food for their herculean tasks. The greater food overtaxes the organs in proportion, and in the same number of years, one animal may live the double of another in usefulness.

We judge the age of an animal, from its general aspect, from the various changes in the conformation of the body, both external and internal, and from the functual activity of its various organs. All of these are to be examined in detail, and the synthesis of the result usually gives us an accurate indication of its age. The most important details in judging of the age, especially in the horse, are found in the teeth. We find the wear and tear of these organs directly in relation to the amount of aliment which they have to handle, and the amount of aliment directly in relation to the number of years which the animal has lived; taking always into consideration that the energetic draft horse and the rapid race horse, which are obliged to do excessive work, will have their organs more rapidly modified by the excessive nature of the work which they are obliged to perform.

In the ox and sheep, the teeth are again of great value in judging of age, but the epidermic product in the shape of the horns are also important factors. In the dog, the teeth are valuable in indicating the age of young animals, but in older ones, we find that the variable mode of life to which these animals are subject, so modify these organs that they are of little value, and in them we must look to alteration of the epidermic appendages of the skin, and to conditions in their general aspect for our best guide.

*Teeth.*—The teeth are described by Cuivier as "mechanical "instruments in the vertebrated animals, at the entrance of the "alimentary canal, designed to seize, cut, tear, bruise and grind "nutritive substances, before their transmission to the mouth, "pharynx and the œsophagus."

Professor Owen says "they present many varieties as to number, size, form, structure, position and mode of attachment, but are principally adapted for seizing, tearing, dividing, pounding and grinding the food. In some species they are modified to serve as formidable weapons of offense and defense; in others as aids in locomotion, means of anchorage, instruments for uprooting or cutting down trees, or for transport and working of building materials."

*In the Horse.*—There are thirty-six or forty teeth according to

FIG. 1.

[Goubaux and Barrier.] Complete detention of the horse. *P* Pincer teeth; *I* Intermediate teeth; *C.* Corner teeth; *T.* Tush or canine teeth; *PM.* Supplementary premolars; *DM.* Deciduous molars; *MP.* Permanent molars.

the sex of the animal, which are divided into three groups. In front the incisors, to each side the tush or canine teeth, and still further back on the sides, the molars. The first are used to grasp and cut the food, the second to tear it, and the third to bruise and grind it up. Following their position in the jaw, the teeth form a parabolic curve, known as the dental arch, of which there are two, one in the upper jaw and one in the lower jaw. These arches are again subdivided into three portions, an anterior and two lateral. The incisor teeth form the anterior part, in the shape of a half circle, convex in front. On each side, directly behind the incisors is a space, larger or smaller according to the sex of the animal, which corresponds to the intermaxillary and maxillary bone, and extends to the lateral part of the dental arch. This is known as the interdental space. It is plain and extensive in mares because they ordinarily do not have tush teeth, but when they do as in the male, it is divided into two parts, known as the anterior and posterior. The latter in the lower jaw is known as the bar of the jaw. Further behind to the right and left, forming, as it were, branches or sides to the dental arch, are found the molar teeth.

In the adult animal there are in each jaw, six incisors, two tush teeth and twelve molars, making a total of forty for the horse, and without the tush teeth thirty-six for the mare. In the colt there are twelve incisors and twelve molars. The latter divided into rows of three above and three below on each side. In the young animal the tush teeth do not exist. However, in the place where they will appear later, we sometimes find small rudimentary teeth with no particular shape. We again sometimes find both in the young, and in the adult animal, a more or less rudimentary premolar tooth, commonly known as *wolf teeth*, which raises the number in the adult animal to forty-four.

*Incisors. a. Incisors of first dentition.*

The milk teeth known also as deciduous or fœtal teeth are twelve in number, six in each jaw, three on each side. The middle ones are known as pincher teeth, the next as the intermediate teeth, and the outside ones as the corner teeth. They are at first imbedded in the body of the bone, and covered by the gum, but when they have protruded from the alveolar cavities, they form a half circle, convex in front. Compared with permanent teeth, they are shorter, they have a constriction in the cente which is known as the neck, which divides them into a free portion or crown, and imbedded portion or root. They are dead white, milky or yellowish white in color. The anterior face of these teeth is convex in both directions and roughened by little parallel, longitudinal ridges and depressions, which however, become worn off and have a smooth and polished surface as the animal becomes older. The posterior face is concave from above to below and slightly concave from above and below and slightly convex from side to side. The internal border of each tooth is thicker than the external. In a virgin tooth (Fig. 5) or one which is not worn by use, the free portion of crown is divided from in front to behind and limited by two borders one anterior *a.* and the other posterior *b.*, which are separated by a cavity known as the cup *c.* The anterior border is the highest and longest, it is shaped convex transversely and is the first portion of the

FIG, 2.

[Goubaux and Barrier] Temporary Incisors, front face.

tooth to come through the gum. The posterior border is lower and appears later, but soon reaches, however, the level of the an-

FIG. 3.

[Goubaux and Barrier] Temporary incisors, profile and table.

erior border from the wearing down of the latter. From the same cause, the cavity or cup which first existed gradually disappears. This is known as the levelling of the teeth. The imbedded portion or root also contains a cavity, known as the internal dental cavity, or pulp cavity *d.* which protects the papilla or pulp of the

FIG. 4.

Goubaux and Barrier. A milk tooth. *A* Posterior face; *B* Anterior face; *C* Profile.

FIG. 5.

[Goubaux and Barrier.] Longitudinal section of a virgin milk tooth.

tooth, but as the animals become older, the teeth elongate by the growth of their imbedded portion, and the internal cavity diminishes in caliber and is nearly obliterated, by the deposit of bony substances; at the same time the bone of the jaw increases in size and the permanent incisors form in them.

These develop behind the deciduous teeth and are at first separated from them by a bony septum, which, however, usually becomes thinned and the permanent tooth pushes the other out, but sometimes they come from an independent opening and the milk tooth remains in place.

FIG. 6.

Goubaux and Barrier. *A* Permanent Incisor ; *B* Deciduous Incisor.

The absorption of the bone, by pressure of the developing teeth, is frequently attended by constitutional phenomena, loss of appetite, sluggishness, etc., and I have seen it produce convulsions in a two-and-a-half year old colt.

[TO BE CONTINUED.]

## Figures and Tables

**Fig. 1. f1:**
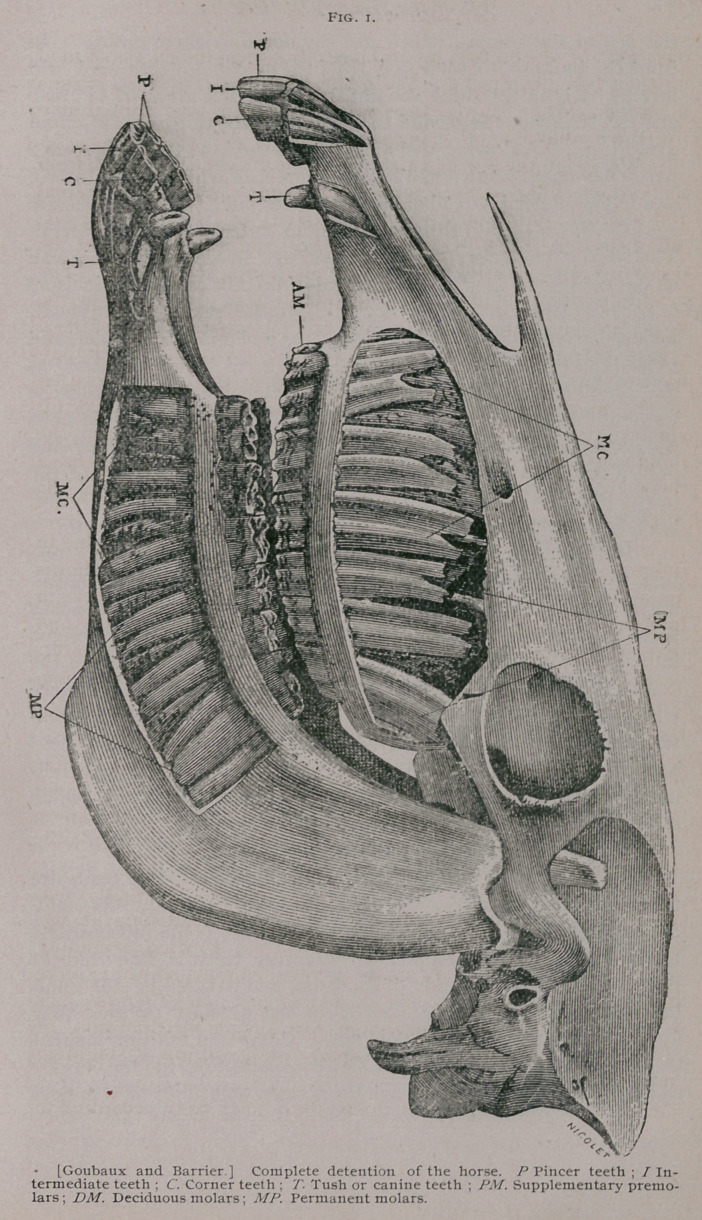


**Fig. 2. f2:**
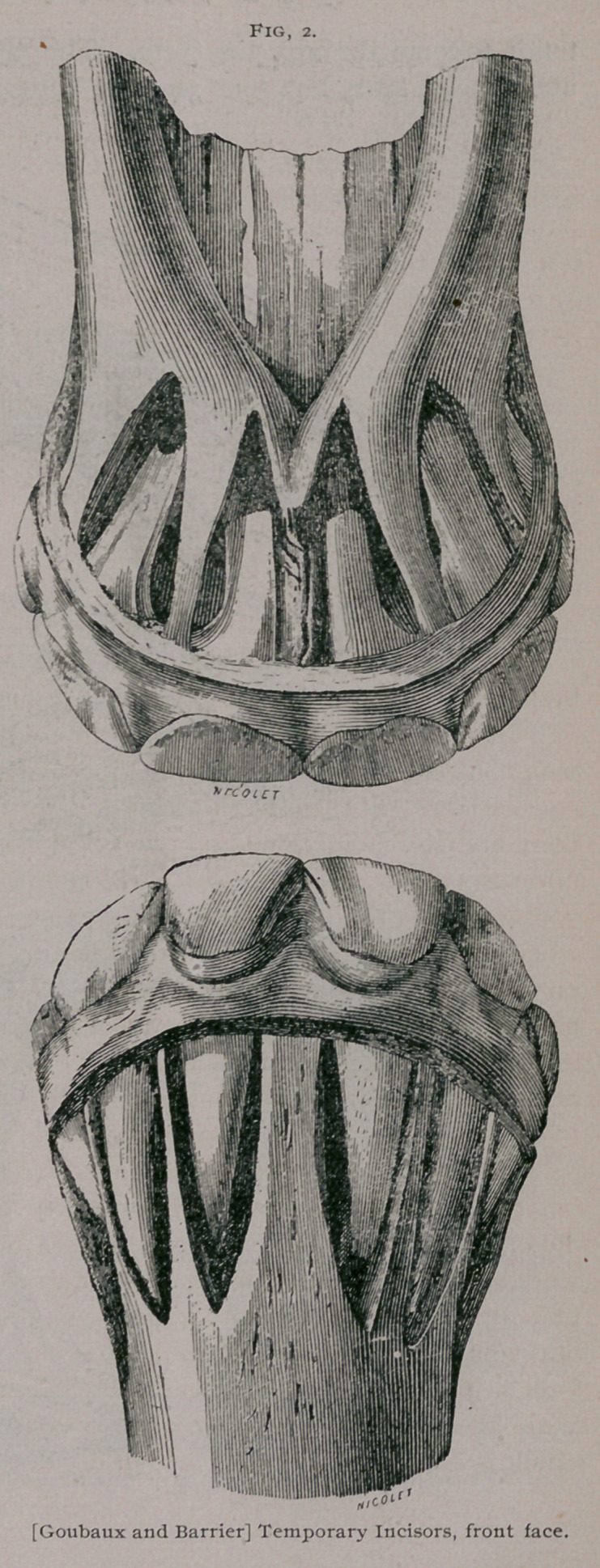


**Fig. 3. f3:**
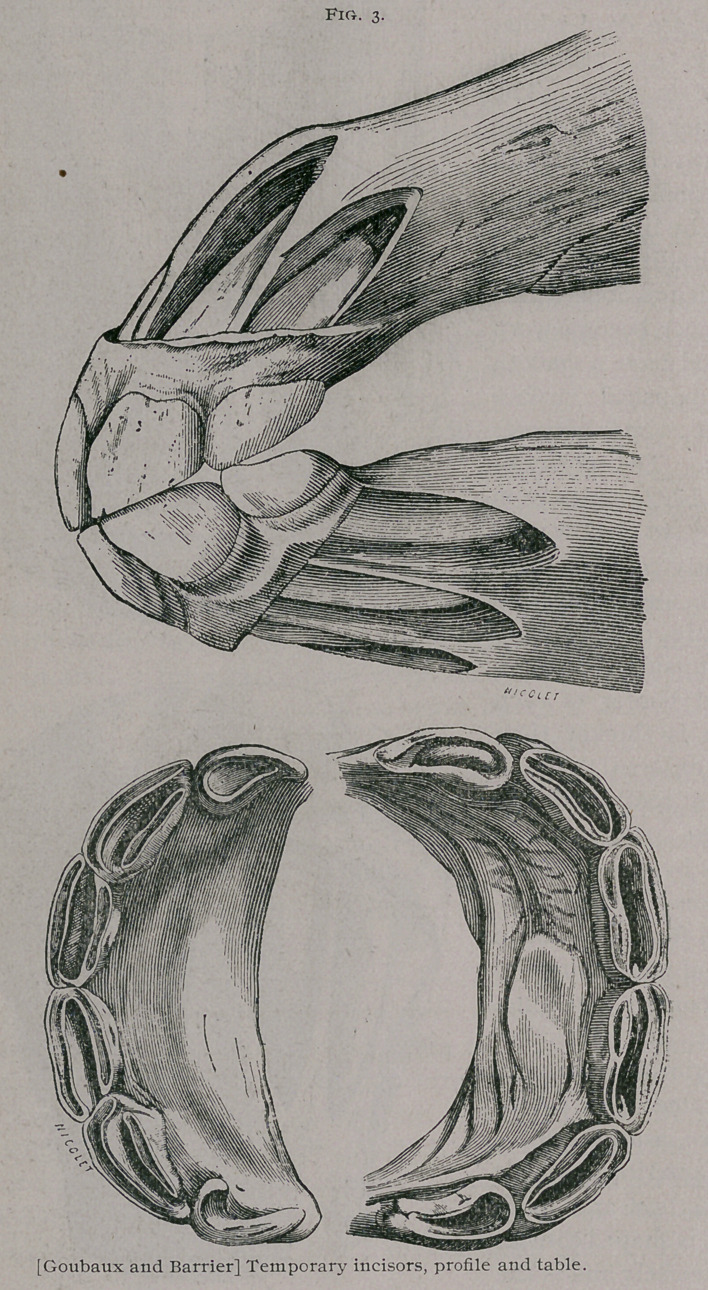


**Fig. 4. f4:**
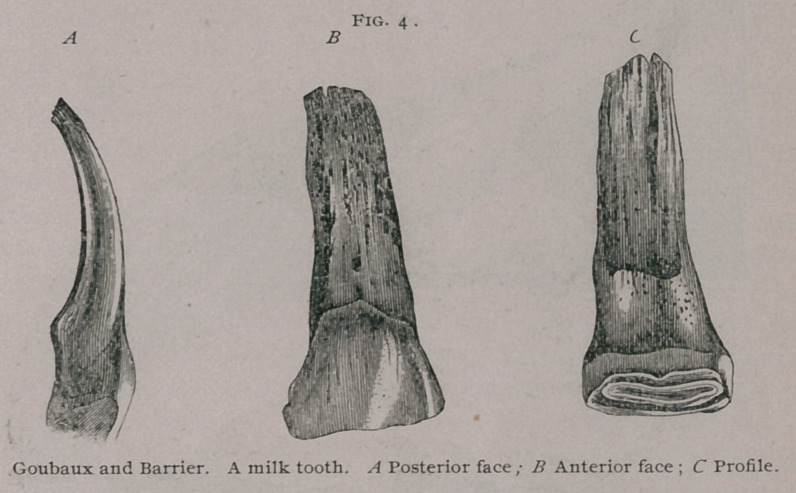


**Fig. 5. f5:**
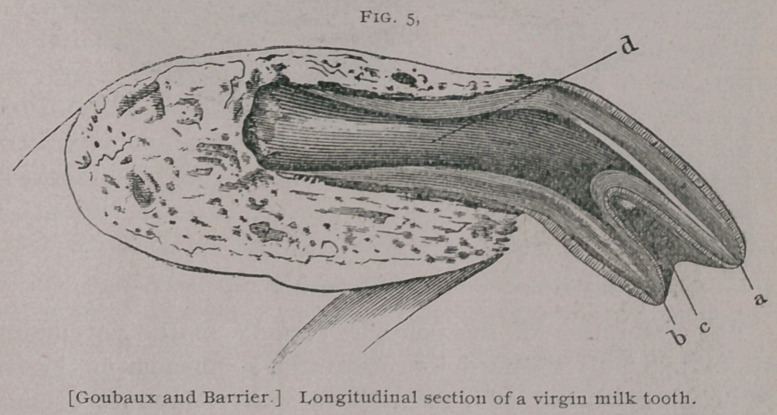


**Fig. 6. f6:**